# A Two-Level Transfer Learning Algorithm for Evolutionary Multitasking

**DOI:** 10.3389/fnins.2019.01408

**Published:** 2020-01-14

**Authors:** Xiaoliang Ma, Qunjian Chen, Yanan Yu, Yiwen Sun, Lijia Ma, Zexuan Zhu

**Affiliations:** ^1^College of Computer Science and Software Engineering, Shenzhen University, Shenzhen, China; ^2^Guangdong Laboratory of Artificial Intelligence and Digital Economy (SZ), Shenzhen University, Shenzhen, China; ^3^National Engineering Laboratory for Big Data System Computing Technology, Shenzhen University, Shenzhen, China; ^4^School of Medicine, Shenzhen University, Shenzhen, China

**Keywords:** evolutionary multitasking, multifactorial optimization, transfer learning, memetic algorithm, knowledge transfer

## Abstract

Different from conventional single-task optimization, the recently proposed multitasking optimization (MTO) simultaneously deals with multiple optimization tasks with different types of decision variables. MTO explores the underlying similarity and complementarity among the component tasks to improve the optimization process. The well-known multifactorial evolutionary algorithm (MFEA) has been successfully introduced to solve MTO problems based on transfer learning. However, it uses a simple and random inter-task transfer learning strategy, thereby resulting in slow convergence. To deal with this issue, this paper presents a two-level transfer learning (TLTL) algorithm, in which the upper-level implements inter-task transfer learning via chromosome crossover and elite individual learning, and the lower-level introduces intra-task transfer learning based on information transfer of decision variables for an across-dimension optimization. The proposed algorithm fully uses the correlation and similarity among the component tasks to improve the efficiency and effectiveness of MTO. Experimental studies demonstrate the proposed algorithm has outstanding ability of global search and fast convergence rate.

## Introduction

In recent years, the development of evolutionary computation has attracted extensive attention. Based on the Darwinian theorem of “Survival of the Fittest” (Dawkins, 2006; Ma et al., 2014a), the population-based evolutionary algorithms (EAs) have been successfully used to solve a wide range of optimization problems (Deb, 2001; Qi et al., 2014; Ma et al., 2018). Multitasking optimization (MTO) problems have emerged as a new interest in the area of evolutionary computation (Da et al., 2016; Gupta et al., 2016a; Ong and Gupta, 2016; Yuan et al., 2016). Inspired by the ability of human beings to process multiple tasks at the same time, MTO aims at dealing with different optimization tasks simultaneously within a single solution framework. MTO introduces implicit transfer learning across different optimization tasks to improve the solving of each task (Gupta and Ong, 2016; Gupta et al., 2016b). If the component tasks in an MTO problem possess some commonalities and similarities, sharing knowledge among these optimization tasks is helpful to solve the whole MTO problems (Bali et al., 2017; Yuan et al., 2017).

Transfer learning is a new machine learning method that has caught increasing attention in recent years (Pan and Yang, 2010; Tan et al., 2017). It focuses on solving the target problem by applying the existing knowledge learned from other related problems (Gupta et al., 2018). In general, the more commonalities and similarities are shared between the source problem and target problem, the more effectively the transfer learning work for them. Multifactorial evolutionary algorithm (MFEA) is the first work to introduce transfer learning into the domain of evolutionary computation to deal with MTO problem (Gupta and Ong, 2016). In MFEA, the knowledge is implicitly transferred through chromosomal crossover (Gupta and Ong, 2016). As a general framework, MFEA uses a simple inter-task transfer learning by assortative mating and vertical cultural transmission with randomness, which tends to suffer from excessive diversity thereby leading to a slow convergence speed (Hou et al., 2017).

To deal with the aforementioned issues of MFEA, this paper proposes a two-level transfer learning (TLTL) framework in MTO. The upper level performs inter-task knowledge transfer via crossover and exploits the knowledge of the elite individuals to reduce the randomness, which is expected to enhance the search efficiency. The lower level is an intra-task knowledge transfer for transmitting information from one dimension to other dimensions within the same optimization task. The two levels cooperate with each other in a mutually beneficial fashion. The experimental results on various MTO problems show that the proposed algorithm is capable of obtaining high-quality solutions compared with the state-of-the-art evolutionary MTO algorithms.

In the rest of this paper, section “Background and Related Work” introduces the background of MTO and MFEA as well as the related work of transfer learning in evolutionary computation. The proposed TLTL algorithm is described in section “Method.” Section “Experimental Methodology” presents the MTO test problems. The comparison results between the proposed algorithm and the state-of-the-art evolutionary multitasking algorithms are shown in section “Results.” Finally, section “Discussion and Conclusion” concludes this work and points out some potential future research directions.

## Background and Related Work

This section introduces the basics of MTO and MFEA, and the related work of Evolutionary MTO.

### Multitasking Optimization

The main motivation of MTO is to exploit the inter-task synergy to improve the problem solving. The advantage of MTO over the counterpart single-task optimization in some specific problems has been demonstrated in the literature (Xie et al., 2016; Feng et al., 2017; Ramon and Ong, 2017; Wen and Ting, 2017; Zhou et al., 2017).

Without loss of generality, we consider a scenario in which *K* distinct minimization tasks are solved simultaneously. The *j*-th task is labeled *T*_j_, and its objective function is defined as *F*_j_(x):*X*_j_→*R*. In such setting, MTO aims at searching the space of all optimization tasks concurrently for $\left\{x_{1}^{{}^{*}},\ldots ,x_{\text{k}}^{{}^{*}}\right\}=\text{argmin}\,\left\{F_{1}\,\left(x_{1}\right),\,\ldots ,\,F_{\text{K}}\,\left(x_{\text{k}}\right)\right\}$, where each $x_{\text{j}}^{{}^{*}}$ is a feasible solution in decision space *X*_j_. To compare solution individuals in the MFEA, it is necessary to assign new fitness for each population member *p*_i_ based on a set of properties as follows (Gupta and Ong, 2016).

#### Definition 1 (Factorial Cost)

The *factorial cost* of an individual is defined as α_ij_ = γδ_ij_ + *F*_ij_, where *F*_ij_ and δ_ij_ are the objective value and the total constraint violation of individual *p*_i_ on optimization task *T*_j_, respectively. The coefficient γ is a large penalizing multiplier.

#### Definition 2 (Factorial Rank)

For an optimization task *T*_j_, the population individuals are sorted in ascending order with respect to the *factorial cost*. The *factorial rank r*_*ij*_ of an individual *p*_i_ on optimization task *T*_j_ is the index value of *p*_i_in the sort list.

#### Definition 3 (Skill Factor)

The *skill factor* τ_i_ of an individual *p*_i_ is the component task on which *p*_i_ performs the best τ_i_ = argmin{*r*_ij_}.

#### Definition 4 (Scalar Fitness)

The *scalar fitness* of an individual *p*_i_ in a multitasking environment is calculated by β_i_ = max{1/*r*_*i1*_,…,1/*r*_iK_}.

### Multifactorial Evolutionary Algorithm

This subsection briefly introduces MFEA (Gupta and Ong, 2016), which is the first evolutionary MTO algorithm inspired by the work (Cloninger et al., 1979). MFEA evaluates a population of *N* individuals in a unified search space. Each individual in the initial population is pre-assigned a dominant task randomly. In the process of evolution, each individual is only evaluated with respect to one task to reduce the computing resource consumption. MFEA uses typical crossover and mutation operators of classical EAs to the population. Elite individuals for each task in the current generation are selected to form the next generation.

The knowledge transfer in MFEA is implemented through assortative mating and vertical cultural transmission (Gupta and Ong, 2016). If two parent individuals assigned to different skill factor are selected for reproduction, the dominant tasks, and genetic material of offspring inherit from their parent individuals randomly. MFEA uses a simple inter-task transfer learning and has strong randomness.

### Evolutionary Multitasking Optimization

Transfer learning is one active research field of machine learning, where the related knowledge in source domain is used to help the learning of the target domain. Many transfer learning techniques have been proposed to enable EAs to solve MTO problems. For example, the cross-domain MFEA, i.e., MFEA, solves multi-task optimization problems using implicit transfer learning in crossover operation. Wen and Ting (2017) proposed a utility detection of information sharing and a resource redistribution method to reduce resource waste of MFEA. Yuan et al. (2017) presented a permutation-based MFEA (P-MFEA) for multi-tasking vehicle routing problems. Unlike the original MFEA using a random-key representation, P-MFEA adopts a more effective permutation-based unified representation. Zhou et al. (2017) suggested a novel MFEA for combinatorial MTO problems. They developed two new mechanisms to improve search efficiency and decrease the computational complexity, respectively. Xie et al. (2016) enhanced the MFEA based on particle swarm optimization (PSO). Feng et al. (2017) developed a MFEA with PSO and differential evolution (DE). Bali et al. (2017) put forward a linearized domain adaptation strategy to deal with the issue of the negative knowledge transfer between uncorrelated tasks. Ramon and Ong (2017) presented a multi-task evolutionary algorithm for search-based software test data generation. Their work is the first attempt to demonstrate the feasibility of MFEA for solving real-world problems with more than two tasks. Da et al. (2016) advanced a benchmark problem set and a performance index for single-objective MTO. Yuan et al. (2016) designed a benchmark problem set for multi-objective MTO that can facilitate the development and comparison of MTO algorithms. Hou et al. (2017) proposed an evolutionary transfer reinforcement learning framework for multi-agent intelligent system, which can adapt to the dynamic environment. Tan et al. (2017) introduced an adaptive knowledge reuse framework across expensive multi-objective optimization problems. Multi-problem surrogates were proposed to reuse knowledge gained from distinct but related problem-solving experiences. Gupta et al. (2018) discussed the recent studies on global black-box optimization via knowledge transfer across different problems, including sequential transfer, multitasking, and multiform optimization. For a general survey of transfer learning, the reader is referred to Pan and Yang (2010).

## Method

**FIGURE 1:**
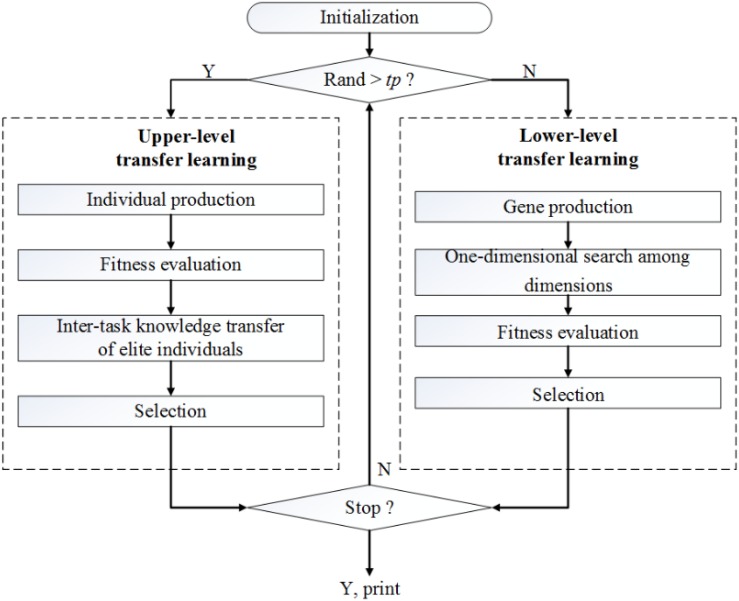
The general flowchart of proposed algorithm.

This section introduces the TLTLA algorithm for MTO. The upper level is an inter-task knowledge learning, which uses the inter-task commonalities and similarities to improve the efficiency of cross-task optimization. The lower level transfer learning focuses on intra-task knowledge learning, which transmits the information from one dimension to other dimensions to accelerate the convergence. The general flowchart of the proposed algorithm is shown in Figure 1.

At the beginning of TLTLA, the individuals in the population are initialized with a unified coding scheme. Let *tp* indicate the inter-task transfer learning probability. If a generated random value is greater than *tp*, the algorithm goes through four steps to complete the inter-task transfer learning process. The parent population produces offspring population by crossover operator and mutate operator. In chromosome crossover, part of the knowledge transfer is realized with the random inheritance of culture and gene from parent to children. However, this pattern is accompanied by strong randomness. To deal with this issue, this paper suggests knowledge transfer of inter-task elite individuals. Finally, the individuals with high fitness are selected into the next generation. If the generated random value is less than *tp*, the algorithm performs a local search based on intra-task knowledge transfer. According to the individual fitness and the elite selection operator, the algorithm executes 1-dimensional search using information from other dimensions. Detailed description of the above two processes are provided in the following subsections.

### Encoding and Decoding

**FIGURE 2:**
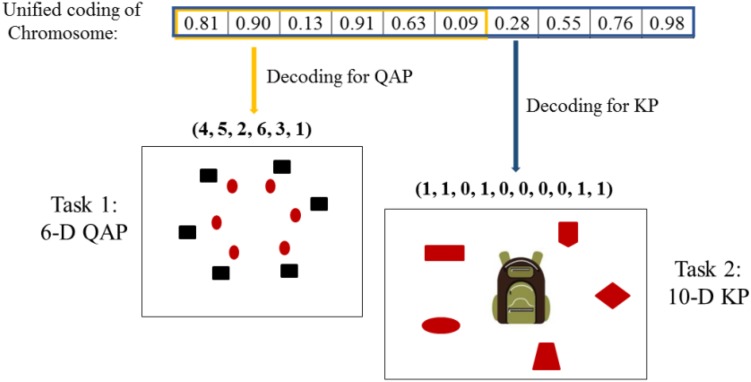
The unified coding and different decoding in multi-tasking optimization with quadratic assignment problem (QAP) and knapsack problem (KP).

To facilitate the knowledge transfer in the multitasking environment, Gupta et al. (2016b) suggested using the unified individual coding scheme. Let *K* denote the number of distinct component tasks in the multitasking environment, the search space dimension of the *i*-th task is denoted as *D*_i_. Through the unified processing, the number of decision variables of every chromosome is set to *D*_MTO_ = max{*D*_i_}. Each decision variable in a chromosome is normalized in the range [0, 1] as shown in Figure 2. Conversely, in the phase of decoding, each chromosome can be decoded into a task-specific solution representation. For the *i*-th task *T*_i_, we extract *D*_i_ decision variables from the chromosome, and decoded these decision variables into a feasible solution for the optimization tasks *T*_i_. In general, the extracted part is the first *D*_i_ decision variables of the chromosome.

### Initialization

**FIGURE 3:**
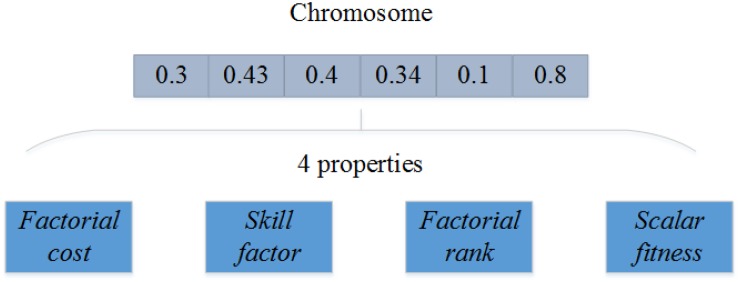
Representation scheme of an individual.

In the initialization, a population *p*_0_ of *N* individuals is generated randomly by using a unified coding scheme. Every individual is encoded in a chromosome and associated with a set of properties including *factorial cost*, *skill factor*, *factorial rank*, and *scalar fitness*. The four properties have been described in section “Background and Related Work.” Representation scheme of an individual is shown in Figure 3.

In such a setting, considering *K* optimization tasks in the initial multitasking environment, we assign the equal computation resource to each component task. In other words, the subpopulation of each component task is composed by *N/K* individuals in the evolutionary process.

### Fitness Evaluation

**FIGURE 4:**
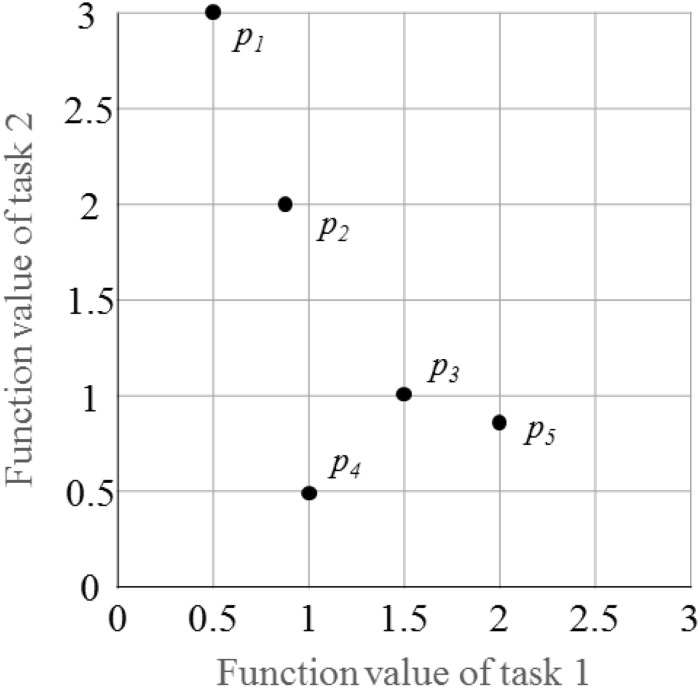
Five points in two-task optimization problem.

In a multitasking environment, an individual may optimize one or multiple optimization tasks. Herein, a generic way is used to calculate the fitness of each individual (Gupta and Ong, 2016). Figure 4 and Table 1 illustrate the fitness assignment of the individuals in a two-task optimization problem.

**TABLE 1:** The results of calculating individual fitness.

**Individual**	**Factorial cost**		**Factorial rank**		**Skill factor**	**Scalar fitness**
	**α_*i1*_**	**α_*i2*_**	***r*_*i1*_**	***r*_*i2*_**	**τ_i_**	**β_i_**
*p*_1_	0.5	3	1	5	*T*_1_	1
***p*_2_**	**0.8**	**2**	**2**	**4**	***T*_1_**	**1/2**
*p*_3_	1.5	1	4	3	*T*_2_	1/3
*p*_4_	1	0.5	3	1	*T*_2_	1
*p*_5_	2	0.8	5	2	*T*_2_	1/2

*Bold values in the case used to explain the concept of individual fitness evaluation in multitasking environment.*

As shown in Figure 4, five individuals and their corresponding fitness function values on different tasks are given. According to the definitions of four properties described in the section “Background and Related Work,” the corresponding values are shown in Table 1. For example, individual *p*_2_ has factorial costs 0.8 and 2 on component tasks *T*_1_ and *T*_2_, respectively. After sorting all individuals based on their factorial costs in ascending order, the factorial ranks of individual *p*_2_ on tasks *T*_1_ and *T*_2_ are 2 and 4, respectively. Thus, the final scalar fitness and skill factor of individual *p*_2_ are 1/2 = max{1/2, 1/4} and *T*_1_, respectively.

### Inter-Task Knowledge Transfer

This subsection describes the inter-task transfer learning in Algorithm 1, which enables the discovery and transfer of existing genetic material from one component task to another. Individuals in the multitasking environment may have different cultural backgrounds, i.e., different skill factors. When the cultural background of an individual is changed, the individual is transferred from one task to another (Gupta and Ong, 2016). One of the drawbacks in MFEA is the strong randomness in its inter-task knowledge transfer. To deal with this issue, an elite individual transfer is proposed in this subsection.

**Algorithm 1:** Inter-task transfer learning.
**Require:**
*Pt*, the current population;
*rmp*, the balance factor between crossover and mutation;
*N*, the population size;
*K*, the number of component tasks.
1.    **for** *i* = 1 to *N*/2 **do**
2.       Randomly choose parents (*pa, pb*) from *P*_t_
3.       **if** (*τa* == *τb*) or (*rand* < *rmp*)
4.          (*ca, cb*) = crossover on (*pa, pb*)
5.          *ca* and *cb* randomly inherits *τa* or *τb*
6. **else**
7.          *ca* = mutation in (*pa*) and *cb* = mutation on (*pb*)
8.          *ca* inherits (*τa*) and *cb* inherits (τb)
9.       **end if**
10. **end for**
11. **for** *i* = 1 to *N* **do**
12.     Evaluate *ci* on task *τi*
13. **end for**
14. Compute *factorial rank* for all individuals
15. Record elite individuals (*factorial rank* == 1) as *Bt* = {*b1*,…,*bK*} and set
16. **for** *i* = 1 **to** *K*
17.         Evaluate *bi* on task *τ_*r*_*, where *r* = *rand* (*K*) and *r* ! = *i*
18.         Put the evaluated individualinto
19. **end for**
20. *R*_t_ = *C*_*t*_ ∪ *P*_*t*_ ∪ *B*${}_{t}^{r}$
21. Compute scalar fitness for all individuals
22. Select N elite individuals from *R*_*t*_ to *P*_*t*__+__1_
23. Set *t* = *t*+1

There are two ways of inter-task individual transfer in Algorithm 1. One is implicit genetic transfer through chromosomal crossover as shown in line 5 (Gupta and Ong, 2016). If two parent individuals with different cultural backgrounds undergo crossover, their offspring can inherit from one of them (Cavallisforza and Feldman, 1973; Gupta and Ong, 2016). The other is the elite individual transfer among tasks, which interchanges the skill factor of the best individuals among tasks in lines 17. If multiple optimization tasks are of commonality and similarities, a good solution to one task is also expected to have a good performance on other tasks. To reduce resource consumption, this operation is applied to the best individuals only.

#### Individual Production

In inter-task transfer learning, the proposed algorithm uses the simulated binary crossover (SBX) (Deb and Agrawal, 1994; Ma et al., 2016b) operator and the polynomial mutation (Ma et al., 2016a) operator to produce the offspring population.

In lines 2–9 of Algorithm 1, assortative mating and vertical cultural transmission are performed in the parent pool. Specifically, two randomly selected parent individuals undergo crossover or mutation based on the balance factor rmp. In the crossover operation, the mating of parent individuals with different skill factor may lead to the emergence of genetic transfer (Cavallisforza and Feldman, 1973; Feldman and Laland, 1996). Each child imitates the skill factor from one of the two parent individuals randomly. The random inheritance mechanism can be considered as an inter-task knowledge transfer, which shares relevant information for promoting population evolution.

#### Inter-Task Knowledge Transfer of Elite Individuals

Due to the strong randomness of assortative mating and vertical cultural transmission, population evolution has some limitations in the global search and convergence. In lines 15–19 of Algorithm 1, an elite individual transfer is introduced to alleviate this issue.

In each generation, the best individual of each component task (i.e., the factorial rank of this individual is 1) is recorded in line 15. Considering the commonalities and similarities among different tasks, a new skill factor for each best individual is assigned and evaluated with respect to the new task. The inter-task knowledge transfer of elite individuals is shown in line 17. If multiple optimization tasks are of strong commonalities and similarities, a good solution of one task is also expected to have good performance on the other tasks.

#### Evaluation and Selection

As shown in line 20, the combined population R_t_ consists of parent population P_t_, offspring population C_t_, and learned individuals $B_{\text{t}}^{\text{r}}$. An elitist selection operator is used and the individuals with higher scalar fitness are selected into the next generation in line 22.

### Intra-Task Knowledge Transfer

Besides, inter-task transfer learning, the proposed algorithm is also characterized with intra-task transfer learning as shown in Algorithm 2. The intra-task transfer learning transmits the knowledge from one dimension to other dimensions within the same task. The proposed cross-dimensional one-dimensional search complements well with SBX and is expected to prevent the algorithm from getting trapped in local optima.

**Algorithm 2:** Intra-task transfer learning.
**Require:**
*P_t_*, the current population;
*S*, the number of variables in unified individual coding.
1. **for** *i* = 1 to *S* **do**
2.    Randomly select an individual *p_r_* from *P_t_*
3.    *Off* (1, *S*) = differential evolution on {*x_i_*}
4.    **for** *j* = 1 to *S* **do**
5.       *d_j_* = (*p_r_* (1),…,*p_r_*(*j-1*), *Off* (*j*), *p_r_* (*j+1*), …, *p_r_* (*S*))
6.       Evaluate *d_j_* on task τ_pr_
7.       **if** *d_j_* is better than *p_r_*
8.          *p_r_*(j) = *Off* (*j*)
9.       **end if**
10.   **end for**
11. **end for**

#### One-Dimensional Mutation

At the beginning of Algorithm 2, an individual is randomly selected from the current population in line 2. In line 3, S offspring genes [Off(1),…,Off(S)] are generated by DE mutation operator (Qin and Suganthan, 2005; Ma et al., 2014b,c), with the parent genes coming from the i-th dimension variable x_i_ of the population.

#### One-Dimensional Search Among Dimensions

**FIGURE 5:**
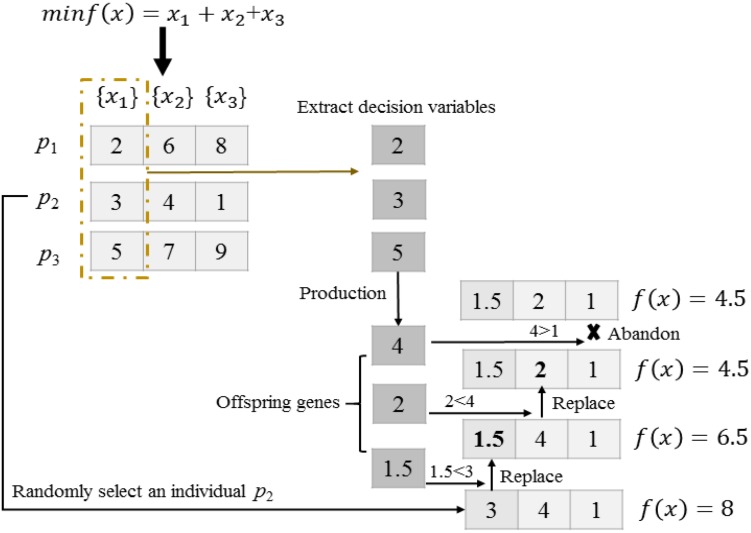
An example of transfer learning among different dimensions.

As shown in lines 4–10 of Algorithm 2, S offspring are iteratively used to compare with the S variables of the selected individual p_r_ as shown in Figure 5. Three individuals with the same dominant task are given in the search space. Firstly, we randomly select an individual p_2_ from the current population. Secondly, three decision variables 2, 3, and 5 are extracted in the 1st dimension of individuals p_1_, p_2_, and p_3_, respectively. Thirdly, three extracted decision variables undergo DE to generate three offspring genes 4, 2 and 1.5. Finally, the cross-dimensional search for individual p_2_ is performed to find out improved solutions. Offspring genes 1.5 and 2 replace the parent genes 3 and 4, respectively, as they obtain better fitness. On the contrary, offspring gene 4 is abandoned as it attains no improvement.

#### Evaluation and Selection

The evaluation and selection of a temporary individual d_j_ constructed by the one-dimensional search are shown in lines 8–11. To reduce the number of function evaluations, the temporary individual d_j_ is evaluated only on task τ_p_r_. In line 7, if the new constructed individual d_j_ is better than p_r_ in terms of fitness value, p_r_ is updated by d_j_ in line 8.

## Experimental Methodology

The proposed TLTLA is compared with the state-of-the-art evolutionary MTO algorithms, i.e., MFDE (Feng et al., 2017), MFEA (Gupta and Ong, 2016), and SOEA (Gupta and Ong, 2016). The benchmark MTO problems (Da et al., 2016) are used to test the algorithms. All test problem are bi-tasking optimization problems. To verify the effectiveness of the compared algorithms, component tasks in MTO problems possess different types of correlation in Da et al. (2016). To demonstrate the scalability of the proposed algorithm on more complex problems, we also construct nine tri-tasking optimization problems in this study.

### Optimization Functions

This section introduces seven elemental single-objective continuous optimization functions (Da et al., 2016) used to construct the MTO test problems. The specific definitions of these seven functions are shown as follows. In particular, the dimensionality of the search space is denoted as D.

(1) Sphere:

$$F_{1}\,\left(x\right)=\sum_{i=1}^{D}x_{i}^{2},x\in {\left[-100,100\right]}^{D}$$

(2) *Rosenbrock:*

$$F_{2}\,\left(x\right)=\sum_{i=1}^{D-1}\left(100\,{\left(x_{i}^{2}-x_{i+1}\right)}^{2}+{\left(x_{i}-1\right)}^{2}\right),x\in {\left[-50,50\right]}^{D}$$

(3) *Ackley:*

$$F_{3}\,\left(x\right)=-20\,\exp\left(-0.2\,\sqrt{\frac{1}{D}\,\sum_{i=1}^{D}x_{i}^{2}}\right)-$$

$$\exp\left(\frac{1}{D}\,\sum_{i=1}^{D}\cos\left(2\,\pi \,x_{i}\right)\right)+20+e,x\in {\left[-50,50\right]}^{D}$$

(4) *Rastrigin*:

$$F_{4}\,\left(x\right)=\sum_{i=1}^{D}\left(x_{i}^{2}-10\,\cos\left(2\,\pi \,x_{i}\right)+10\right),x\in {\left[-50,50\right]}^{D}$$

(5) *Schwefel*:

$$F_{5}\,\left(x\right)=418.9829\times D-\sum_{i=1}^{D}x_{i}\,\sin\left({\left|x_{i}\right|}^{\frac{1}{2}}\right),x\in {\left[-500,500\right]}^{D}$$

(6) *Griewank*:

$$F_{6}\,\left(x\right)=1+\frac{1}{4000}\,\sum_{i=1}^{D}x_{i}^{2}-\prod_{i=1}^{D}\cos\left(\frac{x_{i}}{\sqrt{i}}\right),x\in {\left[-100,100\right]}^{D}$$

(7) *Weierstrass*:

$$F_{7}\,\left(x\right)=\sum_{i=1}^{D}\left(\sum_{k=0}^{k_{\max}}\left[a^{k}\,\cos\left(2\,\pi \,b^{k}\,\left(x_{i}+0.5\right)\right)\right]\right)-D\,\sum_{k=0}^{k_{\max}}$$

$$\left[a^{k}\,\cos\left(2\,\pi \,b^{k}\cdot 0.5\right)\right]\,a=0.5,b=3,k_{\max}=20,x\in {\left[-0.5,0.5\right]}^{D}$$

### Two Multitasking Optimization Problem Sets

The nine bi-tasking optimization problems were first proposed in Da et al. (2016), based on which nine tri-tasking optimization problems are constructed in this paper. The properties of the bi-tasking optimization problems are summarized in Table 2, which clearly shows the commonalities and similarities among component tasks.

**TABLE 2:** Nine bi-tasking benchmark problems.

**Two-task problem**	**Intersection and similarity**	**Task**	***R*_*s*_**	***D*_*T*_**	**Global optimal**	**Landscape**
1	CI+HS	Griewank	1.0000	50	(0, 0, . . ., 0)∈[−100, 100]^50^	Multimodal+Non-separable
		Rastrigin		50	(0, 0, . . ., 0)∈ [−50, 50]^50^	Multimodal+Non-separable
2	CI+MS	Ackley	0.2261	50	(0, 0, . . ., 0)∈ [−50, 50]^50^	Multimodal+Non-separable
		Rastrigin		50	(0, 0, . . ., 0)∈ [−50, 50]^50^	Multimodal+Non-separable
3	CI+LS	Ackley	0.0002	50	(42.0969, . . ., 42.0969)∈ [−50, 50]^50^	Multimodal+Non-separable
		Schwefel		50	(420.9687, . . ., 420.9687)∈ [−500, 500]^50^	Multimodal+Separable
4	PI+HS	Rastrigin	0.8670	50	(0, 0, . . ., 0)∈ [−50, 50]^50^	Multimodal+Non-separable
		Sphere		50	(0, . . ., 0, 20, . . ., 20)∈ [−100, 100]^50^	Unimodal+Separable
5	PI+MS	Ackley	0.2154	50	(0, . . ., 0, 1, . . ., 1)∈ [−50, 50]^50^	Multimodal+Non-separable
		Rosenbrock		50	(1, 1, . . ., 1)∈ [−50, 50]^50^	Multimodal+Non-separable
6	PI+LS	Ackley	0.0725	50	(0, 0, . . ., 0)∈ [−50, 50]^50^	Multimodal+Non-separable
		Weierstrass		25	(0, 0, . . ., 0)∈ [−0.5, 0.5]^25^	Multimodal+Non-separable
7	NI+HS	Rosenbrock	0.9434	50	(1, 1, . . ., 1)∈ [−50, 50]^50^	Multimodal+Non-separable
		Rastrigin		50	(0, 0, . . ., 0)∈ [−50, 50]^50^	Multimodal+Non-separable
8	NI+MS	Griewank	0.3669	50	(10, 10, . . ., 10)∈ [−100, 100]^50^	Multimodal+Non-separable
		Weierstrass		50	(0, 0, . . ., 0)∈ [−0.5, 0.5]^50^	Multimodal+Non-separable
9	NI+LS	Rastrigin	0.0016	50	(0, 0, . . ., 0)∈ [−50, 50]^50^	Multimodal+Non-separable
		Schwefel		50	(420.9687, . . ., 420.9687)∈ [−500, 500]^50^	Multimodal+Separable

For the global optimal solutions of the two component tasks, complete intersection (CI) indicates that the global optima of the two optimization tasks are identical on all variables in the unified search space. No intersection (NI) means that the global optima of the two optimization tasks are different on all variables in the unified search space. Partial intersection (PI) suggests that the global optima of the two tasks are the same on a subset of variables in the unified search space.

The similarity (*R*_*s*_) of a pair of optimization tasks are divided into three categories (Da et al., 2016). According to the Spearmans rank correlation similarity metric [40], *R*_*s*_ < 0.2 indicates low similarity (LS), 0.2 < *R*_*s*_ < 0.8 means medium similarity (MS), and *R*_*s*_ > 0.8 denotes high similarity (HS).

In addition to the above nine bi-tasking optimization problems, this paper attempts to solve tri-tasking optimization problems. Nine constructed tri-tasking optimization problems are shown in Table 3.

**TABLE 3:** The mean and standard deviation of function values obtained by TLTLA and MFEA on nine tri-tasking optimization problems.

**Problem**	**TLTLA**			**MFEA**		
	***T*_1_**	***T*_2_**	***T*_3_**	***T*_1_**	***T*_2_**	***T*_3_**
CI+HS++Ackley (50D)	**0.00E+00** (0)	**0.00E+00** (0)	**1.83E-13** (4.72E-13)	3.36E-01 (0.0650)	2.00E+02 (43.5807)	2.87E+00 (0.5167)
CI+MS++Schwefel (50D)	**2.75E-12** (9.29E-12)	**1.74E+01** (55.6038)	**2.96E+02** (1.32E+03)	5.26E+00 (0.8443)	2.68E+02 (58.3610)	3.77E+03 (497.5763)
CI+LS++Weierstrass (25D)	**9.20E-12** (2.09E-11)	**6.36E-04** (1.11E-19)	**6.64E-01** (1.2568)	2.02E+01 (0.0738)	3.91E+03 (583.5658)	2.03E+01 (2.1087)
PI+HS++Ackley (50D)	**2.20E+01** (46.7283)	**7.85E-04** (0.0030)	**1.45E+00** (0.9410)	2.78E+02 (66.1748)	1.25E+01 (1.7731)	5.24E+00 (1.0121)
PI+MS++Schwefel (50D)	**1.05E+00** (1.0191)	**2.06E+01** (23.1907)	**2.96E+02** (1.32E+03)	3.76E+00 (0.5517)	8.96E+02 (206.5210)	3.94E+03 (413.8822)
PI+LS+ Rastrigin (50D)	**1.74E-12** (7.67E-12)	**1.98E-18** (7.90E-34)	**0.00E+00** (0)	4.91E+00 (1.0324)	5.42E+00 (1.1193)	2.45E+02 (41.2149)
NI+HS++Ackley (50D)	**3.54E+01** (20.1767)	**0.00E+00** (0)	**2.68E-14** (3.43E-14)	5.98E+02 (213.2004)	2.06E+02 (46.6145)	3.60E+00 (0.8252)
NI+MS++Rastrigin (50D)	**3.00E-12** (1.33E-11)	**1.04E-02** (0.0321)	**1.98E+01** (49.8744)	4.74E-01 (0.0784)	2.01E+01 (2.8085)	5.59E+02 (132.9283)
NI+LS++Griewank (50D)	**0.00E+00** (0)	**6.36E-04** (1.11E-19)	**0.00E+00** (0)	2.07E+02 (57.5701)	3.81E+03 (518.0790)	4.58E-01 (0.0671)

## Results

### Experimental Results on Bi-Task Optimization Problems

On the nine bi-tasking optimization problems, the population size is set to *N* = 100 for TLTLA, MFDE, MFEA, and SOEA. The maximum number of function evaluations is set to be 50,000 for SOEA and 100,000 for TLTLA, MFDE, and MFEA. Since SOEA is a single-tasking algorithm, it has to be run twice on bi-tasking problems. As such, SOEA consumes the same computational budget with other algorithms. All compared algorithms are performed in 20 independent runs on each MTO problem. The balance factor between crossover and mutation is set to *rmp* = 0.3 in TLTLA, MFDE, and MFEA.

**FIGURE 6:**
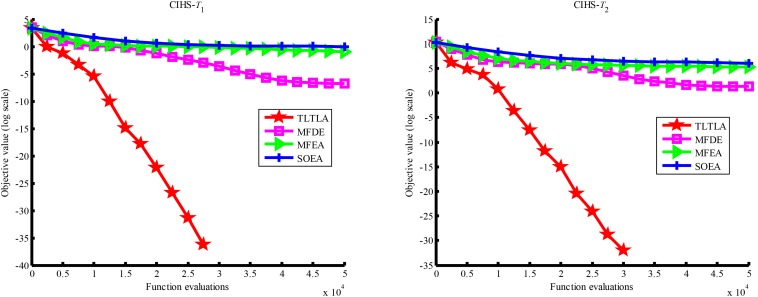
Convergence trends of tasks in CI+HS.

**FIGURE 7:**
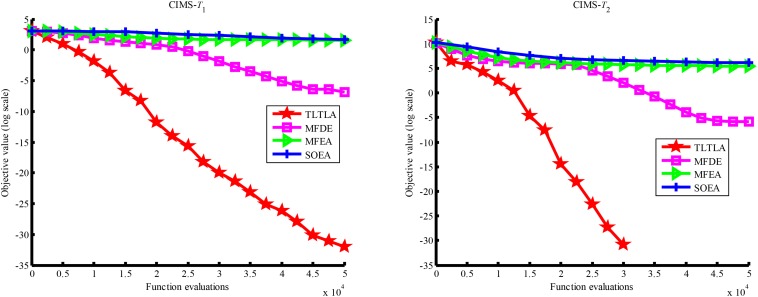
Convergence trends of tasks in CI+MS.

**FIGURE 8:**
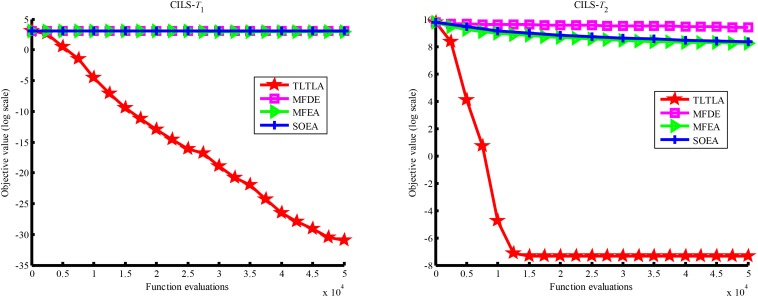
Convergence trends of tasks in CI+LS.

**FIGURE 9:**
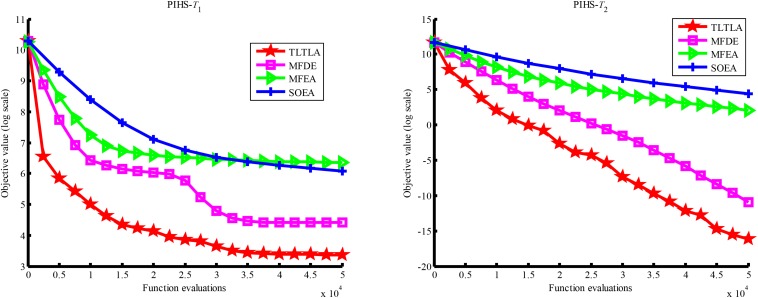
Convergence trends of tasks in PI+HS.

**FIGURE 10:**
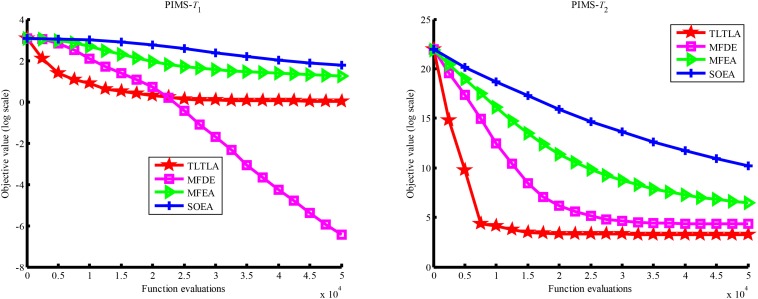
Convergence trends of tasks in PI+MS.

**FIGURE 11:**
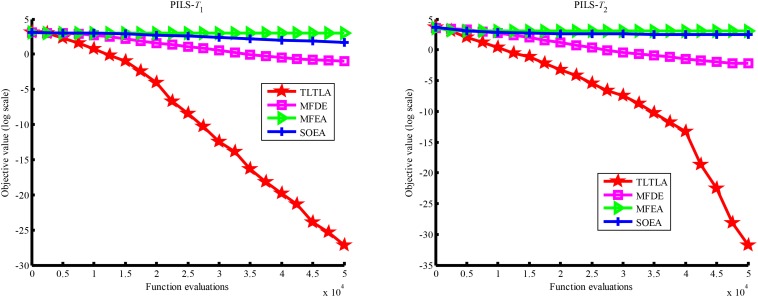
Convergence trends of tasks in PI+LS.

**FIGURE 12:**
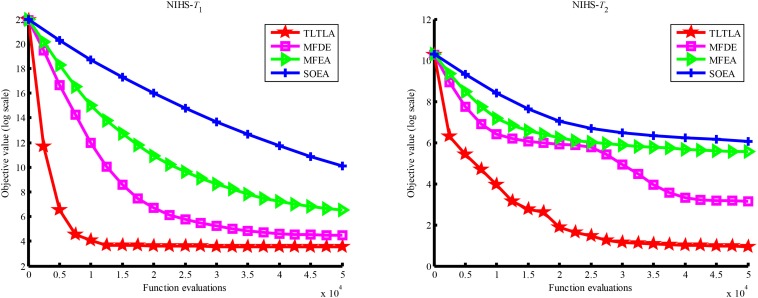
Convergence trends of tasks in NI+HS.

**FIGURE 13:**
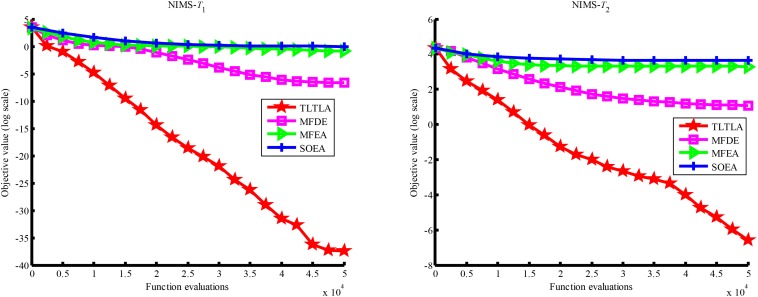
Convergence trends of tasks in NI+MS.

**FIGURE 14:**
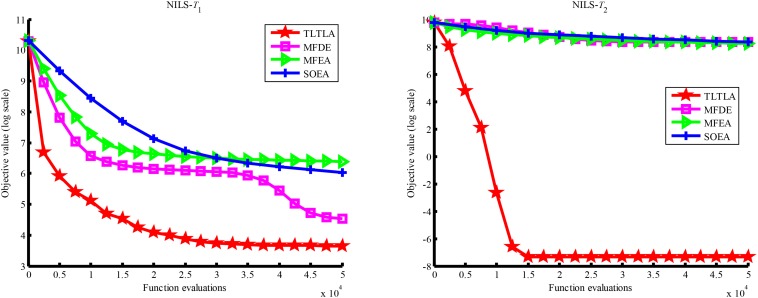
Convergence trends of tasks in NI+LS.

Table 4 presents the mean and standard deviation of function values obtained by the four compared algorithms on nine bi-tasking optimization problems. The best mean function value on each task is highlighted in bold. Compared with MFEA, MFDE and SOEA, TLTLA obtains much better performance. TLTLA obtains the best results in 17 out of 18 independent optimization tasks, except the task *T*_1_ of the PI+MS problem. To study the search efficiency of TLTLA, MFDE, MFEA, and SOEA, Figures 6–14 show the convergence trends of all compared algorithms on the representative optimization tasks. In terms of convergence rate, TLTLA obtains a better overall performance than MFDE, MFEA, and SOEA on most of optimization tasks.

**TABLE 4:** The mean and standard deviation of function values obtained by four compared algorithms on nine bi-tasking optimization problems.

**Problem**	**Task**	**TLTLA**	**MFDE**	**MFEA**	**SOEA**
CI+HS	*T*_1_	**0.00E** + **00**(0)	1.00E−03(3.05E−03)	3.73E−01(0.0617)	9.08E−01(0.0585)
	*T*_2_	**0.00E** + **00**(0)	2.61E + 00(7.96)	1.95E + 02(34.4953)	4.10E + 02(49.0439)
CI+MS	*T*_1_	**1.20**E−14(2.47E−14)	1.00E−03(0.003)	4.39E + 00(0.4481)	5.32E + 00(1.2338)
	*T*_2_	**0.00E** + **00**(0)	3.00E−03(0.012)	2.27E + 02(52.2778)	4.41E + 02(65.0750)
CI+LS	*T*_1_	**3.41E**−**14**(1.21E−14)	2.12E + 01(0.04)	2.02E + 01(0.0798)	2.12E + 01(0.2010)
	*T*_2_	**6.36E**−**04**(1.11E−19)	1.84E + 04(1578.16)	3.70E + 03(429.1093)	4.18E + 03(657.2786)
PI+HS	*T*_1_	**2.88E** + **01**(62.1998)	7.83E + 01(15.37)	6.14E + 02(131.0438)	4.45E + 02(57.2891)
	*T*_2_	**9.63E**−**08**(3.86E−07)	2.20E−05(2.90E−05)	1.01E + 01(2.4734)	8.40E + 01(17.1924)
PI+MS	*T*_1_	1.02E + 00(1.1088)	**1.00E**−**03**(0.001)	3.49E + 00(0.6289)	5.07E + 00(0.4417)
	*T*_2_	**2.65E** + **01**(24.5602)	6.03E + 01(20.53)	7.02E + 02(267.8668)	2.40E + 04(10487.2597)
PI+LS	*T*_1_	**1.60E**−**12**(4.90E−12)	4.60E−01(0.58)	2.00E + 01(0.1302)	5.05E + 00(0.6299)
	*T*_2_	**1.59E**−**14**(6.32E−14)	2.20E−01(0.47)	1.93E + 01(1.7291)	1.32E + 01(2.3771)
NI+HS	*T*_1_	**3.52E** + **01**(20.8321)	8.93E + 01(48.60)	1.01E + 03(346.1264)	2.43E + 04(5842.0394)
	*T*_2_	**2.54E** + **00**(11.3913)	2.05E + 01(15.41)	2.87E + 02(92.4182)	4.48E + 02(61.1642)
NI+MS	*T*_1_	**5.55E**−**17**(2.23E−16)	2.03E−03(0.0042)	4.20E−01(0.0654)	9.08E−01(0.0702)
	*T*_2_	**1.35E**−**03**(0.0030)	2.97E + 00(1.08)	2.71E + 01(2.6883)	3.70E + 01(3.4558)
NI+LS	*T*_1_	**3.85E** + **01**(89.1612)	9.62E + 01(20.02)	6.51E + 02(98.6871)	4.37E + 02(62.6339)
	*T*_2_	**6.36E**−**04**(7.31E−10)	3.94E + 03(730.99)	3.62E + 03(325.0275)	4.14E + 03(524.4335)

On the MTO problems with the high inter-task similarity or complementarity, such as CI+HS, CI+MS, CI+LS, PI+HS, and NI+HS, as shown in Tables 2, 4, TLTLA performs much better than MFEA, MFDE and SOEA in terms of solution quality. In particular, TLTLA obtains the corresponding global optimum 0 on tasks *T*_1_ and *T*_2_ of CI+HS and task *T*_2_ of CI+MS. Three MTO algorithms, i.e., TLTLA, MFEA, and MFDE, work better than the traditional single-task optimization algorithm SOEA thanks to the use of inter-task knowledge transfer. However, the knowledge transfer in MFEA and MFDE is of strong randomness. TLTLA handles this issue by the inter-task elite individual transfer and intra-task cross-dimensional search. The inter-task elite individual transfer is more suitable for MTO problems with CI, i.e., the global optima of two component optimization tasks are identical in the unified search space. The intra-task transfer learning can improve the population diversity and complement well with SBX.

On some MTO problems, the component tasks have different number and/or different kinds of decision variables, such as PI+LS problem. Let one of the component tasks be α-dimensional and the other be β-dimensional (supposing α < β). Therefore, all the individuals in the unified search space are encoded by β decision variables. Using cross-dimensional search, TLTLA is able to utilize the information of the extra β−α decision variables to optimize the α-dimensional component task, which is ignored by the other compared algorithms. This may be the reason TLTLA performs the best on PI+LS problem.

On separable and non-separable optimization tasks, as shown in Tables 2, 4, TLTLA performs well on all separable optimization tasks but not on the non-separable Rosenbrock function. The reason is that Rosenbrock function is fully non-separable problem making the cross-dimensional search of intra-task knowledge transfer inefficient.

### Experimental Results on Tri-Tasking Optimization Problems

To study the scalability of the proposed algorithm in solving more complex tri-tasking optimization problems, we construct nine tri-tasking optimization problems based on the bi-tasking problems (Da et al., 2016). Specifically, nine tri-tasking optimization problems are constructed by adding an additional task into a bi-tasking optimization problem proposed in Da et al. (2016). All compared algorithms are performed in 20 independent runs on each tri-tasking problem. TLTLA is compared with MFEA. Both algorithms are extended to handle tri-tasking problems. The balance factor between crossover and mutation is set to *rmp* = 0.3 for all compared algorithms. The population size is set to *N* = 150 for all compared algorithms. The maximum number of function evaluations is set to 150,000 for all compared algorithms. It is important to note that the experimental settings assign an equal amount of computing resources for each component optimization task in bi-tasking and tri-task optimization problems.

Table 3 reports the mean and standard deviation of the function values obtained by TLTLA and MFEA on nine tri-tasking optimization problems. The best mean function value on each task is highlighted in bold. As can be summarized in Table 3, TLTLA performs significantly better than MFEA in dealing with the tri-tasking problems. The experimental results in Tables 3, 4 demonstrate the high scalability of the proposed algorithm. When the number of component tasks is increased, TLTLA can still obtain solutions of high quality. In particular, on task *T*_2_ of NI+HS+Ackley and task *T*_1_ of NI+LS+Griewank, the proposed algorithm gets more improvements in solving tri-tasking problem than the corresponding bi-tasking problem. The reason is that the corresponding global optimum 0 of the added Griewank task is found, which indicates that TLTLA can utilize the population diversity in the multitasking environment to escape from the local optima.

### The Effectiveness Analysis of Two Proposed Knowledge Transfers

In this section, we empirically study the effectiveness of the two proposed knowledge transfer methods, including inter-task and intra-task knowledge transfers. Two variants of TLTLA, namely TLTLA-U and TLTLA-L are designed to compared with TLTLA. The former is the same as TLTLA without using the intra-task knowledge transfer, the latter is TLTLA without using the inter-task knowledge transfer. MFEA is also involved in the comparison as the baseline. Table 5 shows the mean and standard deviation of the function values obtained by each compared algorithm on nine bi-tasking optimization problems. The best mean function value on each task is highlighted in bold. The sums of rankings of the four compared algorithms are also presented.

**TABLE 5:** The mean and standard deviation of function values between the algorithms TLTLA, TLTLA-U, TLTLA-L, and MFEA.

**Problem**	**Task**	**TLTLA**	**Rank**	**TLTLA-U**	**Rank**	**TLTLA-L**	**Rank**	**MFEA**	**Rank**
CIHS	*T*_1_	**0.00E+00** (0)	**1**	3.38E-01 (0.0701)	3	7.93E-02 (0.0311)	2	3.73E-01 (0.0617)	4
	*T*_2_	**0.00E+00** (0)	**1**	1.75E+02 (51.3951)	2	5.49E+02 (39.1071)	4	1.95E+02 (34.4953)	3
CIMS	*T*_1_	**1.20E-14** (2.47E-14)	**1**	5.35E+00 (0.9860)	3	2.10E+01 (0.1022)	4	4.39E+00 (0.4481)	2
	*T*_2_	**0.00E+00** (0)	**1**	2.33E+02 (60.9264)	3	5.44E+02 (49.9483)	4	2.27E+02 (52.2778)	2
CILS	*T*_1_	**3.41E-14** (1.21E-14)	**1**	2.01E+01 (0.0431)	2	2.11E+01 (0.0457)	4	2.02E+01 (0.0798)	3
	*T*_2_	**6.36E-04** (1.11E-19)	**1**	3.65E+03 (435.9930)	3	1.91E+00 (1.4314)	2	3.70E+03 (429.1093)	4
PIHS	*T*_1_	**2.88E+01** (62.1998)	**1**	6.80E+02 (165.2077)	4	5.44E+02 (39.4790)	2	6.14E+02 (131.0438)	3
	*T*_2_	**9.63E-08** (3.86E-07)	**1**	7.07E+00 (1.6748)	2	8.99E+00 (4.8763)	3	1.01E+01 (2.4734)	4
PIMS	*T*_1_	1.02E+00 (1.1088)	**1**	3.27E+00 (0.4646)	2	2.09E+01 (0.0578)	4	3.49E+00 (0.6289)	3
	*T*_2_	**2.65E+01** (24.5602)	**1**	6.43E+02 (580.1922)	3	2.60E+02 (46.9642)	2	7.02E+02 (267.8668)	4
PILS	*T*_1_	**1.60E-12** (4.90E-12)	**1**	1.99E+01 (0.1446)	2	2.10E+01 (0.1169)	4	2.00E+01 (0.1302)	3
	*T*_2_	**1.59E-14** (6.32E-14)	**1**	2.08E+01 (3.0661)	3	2.26E+01 (1.8860)	4	1.93E+01 (1.7291)	2
NIHS	*T*_1_	**3.52E+01** (20.8321)	**1**	1.06E+03 (1.20E+03)	4	2.72E+02 (40.9484)	2	1.01E+03 (346.1264)	3
	*T*_2_	**2.54E+00** (11.3913)	**1**	2.58E+02 (90.7596)	2	5.28E+02 (38.6019)	4	2.87E+02 (92.4182)	3
NIMS	*T*_1_	**5.55E-17** (2.23E-16)	**1**	3.76E-01 (0.0754)	3	6.74E-02 (0.0172)	2	4.20E-01 (0.0654)	4
	*T*_2_	**1.35E-03** (0.0030)	**1**	2.76E+01 (2.6969)	3	5.55E+01 (2.3183)	4	2.71E+01 (2.6883)	2
NILS	*T*_1_	**3.85E+01** (89.1612)	**1**	6.52E+02 (120.3008)	4	5.42E+02 (34.9702)	2	6.51E+02 (98.6871)	3
	*T*_2_	**6.36E-04** (7.31E-10)	**1**	3.70E+03 (613.1705)	4	1.93E+00 (1.6964)	2	3.62E+03 (325.0275)	3
SUM			**18**		52		55		55

In Table 5, using only one knowledge transfer method, TLTLA-U and TLTLA-L achieve similar overall performance to MFEA. However, combining two proposed knowledge transfers, TLTLA performs much better than MFEA, TLTLA-U, and TLTLA-L on nine test problems, which indicates that the inter-task and the intra-task knowledge transfer procedures cooperate with each other in a mutually beneficial fashion. Therefore, the inter-task and intra-task transfer learning components are indispensable for the proposed algorithm.

## Discussion and Conclusion

In this paper, a novel evolutionary MTO algorithm with TLTL is introduced. Particularly, the upper level transfer learning uses the commonalities and similarities among tasks to improve the efficiency and effectiveness of genetic transfer. The lower level transfer learning focuses on the intra-task knowledge learning, which transmits the beneficial information from one dimension to other dimensions. The intra-task knowledge learning can effectively use decision variables information from other dimensions to improve the exploration ability of the proposed algorithm. The experimental results on two-task and three-task optimization problems show the superior performance and high scalability of the proposed TLTLA.

Evolutionary MTO is a recent paradigm introducing the transfer learning of machine learning into the evolutionary computation (Zar, 1972; Noman and Iba, 2005; Chen et al., 2011; Zhu et al., 2011, 2015a,b,c, 2016, 2017; Gupta and Ong, 2016; Hou et al., 2017). There remain many open challenging problems. For instance, how to avoid the negative transfer? Most evolutionary MTO algorithms were proposed based on the inter-task similarity and commonality. However, on problems with few inter-task similarity and commonality, these algorithms may have worse performance than those with no transfer learning. To deal with this issue, introducing similarity measurement between two tasks could be a good choice. Moreover, how to extend the existing transfer learning based optimization algorithms to solve large-scale multitask problems in real applications remains a challenging problem.

## Data Availability Statement

The code of the proposed algorithm for this study is available on request to the corresponding author.

## Author Contributions

QC and XM performed the experiments, analyzed the data, and wrote the manuscript with supervision from ZZ, YS, and YY. LM contributed substantially to manuscript revision, editing for language quality, and gave suggestions on experimental studies. All authors provided the critical feedback, edited, and finalized the manuscript.

## Conflict of Interest

The authors declare that the research was conducted in the absence of any commercial or financial relationships that could be construed as a potential conflict of interest.
